# Neuroglia Alterations in the Olfactory Bulbs in Patients with Schizophrenia: An Exploratory Postmortem Study

**DOI:** 10.3390/life16071053

**Published:** 2026-06-24

**Authors:** Artyom Malkov, Alexandra Proshchina, Yuliya Krivova, Anastasia Kharlamova, Olga Godovalova, Victoria Gulimova, Sergey Saveliev

**Affiliations:** 1 FSBSI “Petrovsky National Research Center of Surgery”, 3 Tsyurupy, 117418 Moscow, Russia; proshchina@yandex.ru (A.P.); homulkina@rambler.ru (Y.K.); grossulyar@gmail.com (A.K.); asinello@gmail.com (O.G.); gulimova@yandex.ru (V.G.); embrains@mail.ru (S.S.); 2 Medical and Biological Physics Department, I.M. Sechenov First Moscow State Medical University (Sechenov University), 8-2 Trubetskaya St., 119991 Moscow, Russia

**Keywords:** olfactory bulb, schizophrenia, MBP, GFAP, oligodendroglia, astroglia

## Abstract

The human olfactory bulb is a promising structure for the investigation of central nervous system disorders, including dementias of various etiologies. In Alzheimer’s disease, anosmia is among the earliest clinical manifestations. Although olfactory disturbances have also been reported in schizophrenia, alterations within the olfactory system remain insufficiently studied. The aim of this study was to identify changes in the olfactory bulbs of patients with schizophrenia. Olfactory bulbs obtained from patients with schizophrenia (n = 23) and individuals without identified nervous system pathology (n = 23) were examined. Patients with schizophrenia demonstrated a statistically significant decrease in the immunoreactive area of myelin basic protein and a significant increase in the immunoreactive area of glial fibrillary acidic protein compared with the control group. In addition, the thickness of the layer of incoming olfactory nerve fibers was significantly reduced in the schizophrenia group. Overall, our findings demonstrate neuroglial alterations in the human olfactory bulb in schizophrenia. Together with observations from other brain regions, these results may indicate that the identified changes are systemic rather than localized in nature.

## 1. Introduction

Impaired olfactory function is often among the earliest symptoms associated with a broad spectrum of psychiatric, neurodegenerative, and infectious disorders [1]. Several nervous system disorders are characterized by symptoms that overlap with those observed in schizophrenia, including the cognitive and emotional-behavioral impairments associated with Alzheimer’s disease and Parkinson’s disease. Notably, anosmia is also a common early symptom of these conditions.

The primary olfactory center of the central nervous system is the olfactory bulb (OB) [2]. The olfactory bulbs are involved in the initial processing of olfactory stimuli. Six main layers are distinguished in the human olfactory bulb: the olfactory nerve fiber layer, the glomerular layer, the external plexiform layer, the mitral cell layer, the internal plexiform layer, and the granular cell layer [3,4]. The first layer is composed of the afferent fibers of olfactory neurons. These fibers form synapses with mitral and periglomerular cells in the glomerular layer. Together, these structures form glomeruli. The external plexiform layer consists primarily of the dendrites of mitral and tufted cells, along with the apical dendrites of granular cells. The next layer is formed mainly by the cell bodies of mitral cells, which may exceed 20 μm in size. The internal plexiform layer is composed predominantly of the axons of mitral and tufted cells. Tufted cells are currently classified into three subgroups: external, middle, and internal tufted cells. The deepest layer consists primarily of the cell bodies of granular cells, which function as inhibitory interneurons. Studies in rodents have shown that these layers also contain other cell types (e.g., Van-Gehuchten, multipolar, and large short-axon cells), which were not the focus of the present study. Nevertheless, these cells constitute an important component of the olfactory bulb neural networks [5,6,7].

The olfactory bulb is a brain structure that exhibits substantial neuroplasticity due to the continuous formation of new neural connections within the glomerular layer, driven by the lifelong renewal of the olfactory epithelium. Studies in rodents have indicated the presence of neurogenesis in this structure; however, evidence regarding its occurrence in humans remains inconclusive [8,9,10,11]. Furthermore, the olfactory bulb serves as one of the major hubs of the glymphatic system, whose precise functional mechanisms have yet to be fully elucidated. Various pathological processes within the central nervous system may be closely associated with impairments in this waste clearance pathway [12]. Consequently, the human olfactory bulb has emerged as a promising target for investigating structural abnormalities associated with psychiatric disorders. Although overt clinical symptoms such as anosmia are not pathognomonic for schizophrenia, subtle olfactory deficits are frequently reported in affected individuals [13,14,15,16,17]. However, because these sensory alterations rarely receive attention during routine psychiatric evaluations, comprehensive post-mortem morphological investigations of the olfactory pathways have historically been limited. This lack of structural data represents a notable gap in the literature, particularly given that the olfactory bulb is an integral component of the limbic system, a key neural network whose dysfunction has been strongly implicated in the pathogenesis of schizophrenia [18,19].

Schizophrenia is a heterogeneous psychiatric disorder characterized by a lifelong course that frequently results in patient disability. It is marked by the presence of both positive symptoms (an excess or distortion of normal functioning, including primarily delusions and auditory or visual hallucinations) and negative symptoms (a deficit or loss of normal functions, encompassing clinical manifestations such as avolition [loss of motivation], anhedonia [inability to experience pleasure], alogia [poverty of speech], affective flattening, and social withdrawal). These symptoms substantially impair the quality of life of both affected individuals and those around them [20]. Moreover, despite numerous morphological [21,22,23], neuroimaging [24,25,26], genetic [27,28,29,30], epidemiological [31,32] and clinical studies [33,34,35], the precise etiology and pathogenesis of the disorder remain unclear [36]. Schizophrenia is currently widely conceptualized as a disorder of integration processes resulting from neural network dysfunction [37].

An important component of neural networks is the glial microenvironment. Based on their morphology and location, several types of astrocytes are distinguished, including protoplasmic, fibrous, and region-specific subtypes. Astrocytes perform a range of neuroprotective, homeostatic, structural, and metabolic functions [38]. In addition, they exert a modulatory influence on the functioning of neural networks [39].

Glial fibrillary acidic protein (GFAP) is a cytoskeletal protein with a molecular weight of approximately 51 kDa and is widely used as a marker of mature astrocytes in the central nervous system [40]. Elevated GFAP levels in cerebrospinal fluid are used as a marker of axonal degeneration following traumatic brain injury [41]. Gliosis is associated with a broad spectrum of central nervous system disorders [42,43]; however, its presence and role in the pathophysiology of schizophrenia remain highly debated [44]. Existing post-mortem studies have produced mixed findings. While several investigations have reported evidence of localized reactive gliosis in specific cortical and subcortical regions, other morphometric studies have demonstrated significant reductions in glial cell density and total glial cell numbers in different brain areas [22,45,46]. Findings related to astrocytes are particularly controversial, as astrogliosis has been demonstrated in some studies [47,48,49,50] but not in others [51,52]. In contrast, evidence regarding oligodendrocytes is more consistent, with a significant reduction in the oligodendroglial population being reported in schizophrenia across multiple investigations [53].

Oligodendrocytes are an important cell type within the central nervous system because they form axonal sheaths and perform several essential functions. In addition to their primary role in myelination, they contribute to synaptic plasticity [54,55], provide neurotrophic support, and help maintain the integrity of the blood–brain barrier under pathological conditions [56,57,58]. A key component of myelin and a principal marker of oligodendrocytes is myelin basic protein (MBP).

MBP is synthesized by oligodendrocytes and is represented by several isoforms [41]. MBP levels in peripheral blood increase following brain injury and in demyelinating diseases. Elevated MBP mRNA expression has also been reported in patients with schizophrenia [59], and increased IgG-mediated hydrolytic activity against MBP has been observed in patients with the paranoid form of schizophrenia and in those experiencing the acute phase of the disease. These findings may indicate the presence of neuroinflammatory demyelinating processes [60]. In contrast, antipsychotic treatment has been associated with a reduction in this activity [61]. Some studies have reported no significant differences in cerebrospinal fluid MBP levels in patients with schizophrenia; however, this measure is not considered particularly informative [62]. Nevertheless, most studies have demonstrated a reduction in oligodendroglial and myelin-associated markers in brain tissue of patients with schizophrenia [45,63,64,65,66].

Oligodendroglial deficiency is considered a common and disease-specific feature of neurodegenerative disorders, as well as an important aspect of cellular pathology in schizophrenia [22,67]. In contrast, alterations in the astroglial population remain less clearly defined. Abnormalities in glial cell composition may disrupt neurotransmitter homeostasis, trophic support, and synaptic plasticity, ultimately leading to impaired synaptic signal transmission.

The aim of this study was to identify alterations in the olfactory bulbs of patients with schizophrenia through an analysis of glial marker expression within the olfactory bulbs.

## 2. Materials and Methods

The study was conducted using autopsy-derived human olfactory bulbs obtained from the collection of the Laboratory of Nervous System Development at the Avtsyn Research Institute of Human Morphology. The material was collected between 2007 and 2012. All procedures were performed in strict accordance with the legislative requirements of the Russian Federation and the relevant versions of the Declaration of Helsinki and were approved by the appropriate ethics committees. The study was also approved by the Institute’s Ethics Committee (Protocol No. 33(9), dated 7 February 2022). All data are presented in anonymized form in accordance with the principle of medical confidentiality.

The samples were divided into two demographically matched groups: a schizophrenia group (n = 23) aged 47–82 years and a control group consisting of individuals without identified nervous system pathology (n = 23) aged 45–84 years. In the schizophrenia group, 11 individuals were middle-aged (45–59 years), 5 were elderly (60–74 years), and 7 were old-aged (75–89 years). In the control group, 11 individuals were middle-aged, 6 were elderly, and 6 were old-aged. Statistical analysis confirmed that the median age did not differ significantly between the schizophrenia group (median = 67, IQR = 55–76) and the control group (median = 68, IQR = 51–75; *p* = 0.61, Mann–Whitney U test). A total of 46 human olfactory bulb samples were examined, including 20 from male and 26 from female subjects. The male-to-female ratio was 9:14 in the schizophrenia group and 11:12 in the control group, with no statistically significant differences in sex distribution between the groups (*p* = 0.76, Fisher’s exact test).

Detailed information on the patients, including sex, age, clinical characteristics, and cause of death, is provided in Table 1. Information regarding olfactory function and the presence of smoking habits was not available.

**Scheme 1:**
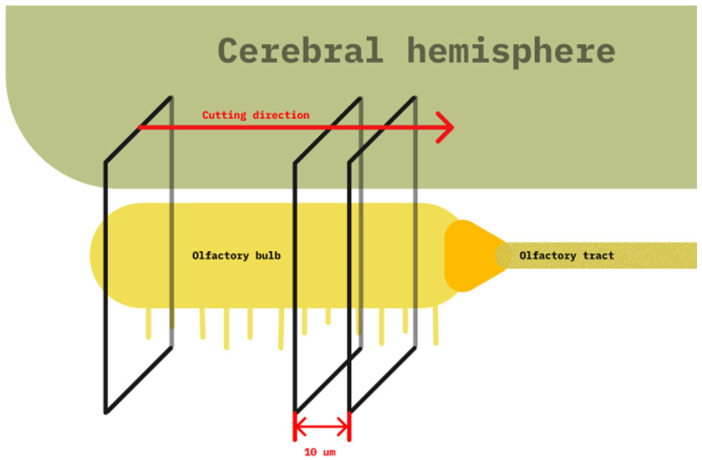
Sectioning of the human olfactory bulb.

The tissue was fixed in 10% neutral buffered formalin (pH 7.0–7.4), followed by dehydration in a series of increasingly concentrated alcohols and dioxane. The olfactory bulbs were then entirely embedded in paraffin blocks (using pure histological paraffin with a melt point of 52–54 °C, Labico LLC, St. Petersburg, Russia), and serial sections 10 micrometers thick were prepared along the entire length of the olfactory bulb in the frontal projection (Scheme 1). The sections were mounted on microscope slides, one section from the middle of each sample for all types of staining described below.

The specimens were stained using the classical histological method with toluidine blue (Nissl staining) for cytoarchitectonic analysis and verification of the olfactory bulb layers.

Chromogenic immunohistochemical staining was performed using primary antibodies against GFAP (Thermo Fisher Scientific Inc., Waltham, MA, USA; mouse monoclonal antibody, Cat# 14-9892-82, RRID: AB_10598206) at a dilution of 1:300 and MBP (Lab Vision, Fremont, CA, USA; rabbit polyclonal antibody, Cat# RB-1460-A0, RRID: AB_60311) at a dilution of 1:100.

For immunohistochemical analysis using anti-GFAP antibodies alone (single staining), the sections were deparaffinized, and endogenous peroxidase activity was blocked by incubation with H_2_O_2_ Block (Thermo Fisher Scientific Inc., USA) for 10 min. The slides were washed in phosphate-buffered saline (PBS; 0.01 M, pH 7.3–7.5; BioLot, St. Petersburg, Russia) after each step. Antigen retrieval was then performed by boiling the sections in citrate buffer (pH 6.0; DiaGene, Mytishchi, Moscow Oblast, Russia) for 10 min, followed by cooling for 20 min. To reduce nonspecific background staining, the sections were incubated with Ultra V Block (Thermo Fisher Scientific Inc., USA) for 7 min. Subsequently, without washing in PBS, the sections were incubated with the primary antibodies for 2 h at room temperature. Visualization was performed using the N-Histofine detection system (Nichirei, Tokyo, Japan) according to the manufacturer’s recommendations.

For double chromogenic immunohistochemical staining, the sections were similarly deparaffinized, endogenous peroxidase activity was blocked, and the slides were washed after each step in Tris-HCl buffer containing 0.1% Tween-20 (TBS Tween-20 Buffer; Thermo Fisher Scientific Inc., USA). Subsequently, following the procedure described above, the sections were prepared up to the stage of primary antibody application and incubated overnight at +4 °C in a humidified chamber. Visualization was performed using reagents from the Multi Vision Polymer Detection System Anti-Mouse HRP + Anti-Rabbit AP, LVblue & LVRed kit (Thermo Fisher Scientific Inc., USA) according to the manufacturer’s recommendations. This staining procedure resulted in red labeling of GFAP and blue labeling of MBP.

In all experiments, negative controls were included for each sample by replacing the primary antibodies with PBS in the single-staining procedure and with TBST in the double chromogenic staining procedure.

The slides were scanned using a KF-PRO-120-HI histoscanner (KFBio, Ningbo, China) equipped with a 20× objective (Olympus UPLXAPO20X, NA 0.8). The thickness of the first olfactory bulb layer was measured on whole-slide scans using Aperio ImageScope (version 12.4.6.) software (Leica Biosystems, Nussloch, Germany) with the “Pen Tool” and “Distance Measurement” functions. For single staining, 20 measurement points were evenly distributed along the entire perimeter of the bulb, and the mean value was calculated. The areas of immunopositive staining and the total section areas were analyzed using ImageJ (version 1.54p) [68,69] under the supervision of two operators.

In the case of single chromogenic immunohistochemical staining for GFAP, the thickness of the first layer (the layer of incoming neuronal fibers) was measured in the control (C) and schizophrenia (SZ) groups, followed by stratification into age subgroups.

Statistical analysis was performed using Statistica 10 (StatSoft, Inc., Tulsa, OK, USA). Data normality was assessed using the Shapiro–Wilk test because the sample size did not exceed 50 observations. Normal P-plots were additionally constructed to verify the distribution of the data, and Levene’s test was used to assess the homogeneity of variances between independent samples.

For the comparison of first-layer thickness between the schizophrenia and control groups, data normality was evaluated using the Shapiro–Wilk test. The null hypothesis of a normal distribution was rejected for both groups (*p* = 0.01 and *p* = 0.044, respectively). Consequently, the non-parametric Mann–Whitney U test was used for group comparisons.

When normality was assessed within the age subgroups, the hypothesis of a normal distribution was rejected only for the middle-aged schizophrenia subgroup based on the Shapiro–Wilk test (*p* = 0.019). For all other subgroups, *p* > 0.05. However, the subgroup data did not satisfy the assumption of homogeneity of variances. In addition, the Normal P-Plot demonstrated a systematic deviation of lower values from the expected normal distribution. Consequently, the non-parametric Kruskal–Wallis ANOVA followed by the post hoc Multiple Comparisons of Mean Ranks test was used for the analysis.

The area of immunopositive staining relative to the total section area was then calculated for GFAP and MBP. In addition, the ratio of the GFAP+/MBP+ area to the total section area was determined in the double-stained sections. The normality of data distribution was assessed using the Shapiro–Wilk test. Because the null hypothesis of a normal distribution was rejected for several variables, non-parametric statistical methods were applied. Comparisons between the two primary cohorts were performed using the Mann–Whitney U test. For multiclass analyses involving age-related subgroups, intergroup differences were evaluated using the Kruskal–Wallis ANOVA followed by the post hoc Multiple Comparisons of Mean Ranks test, which inherently adjusts calculated *p*-values for multiple comparisons. To assess the relationship between GFAP+ staining area and patient age, Spearman’s correlation coefficient was used because of the non-normal data distribution.

Results are presented as the median (Me) and quartiles (Q1; Q3). Differences were considered statistically significant at *p* < 0.05.

## 3. Results

**Figure 1:**
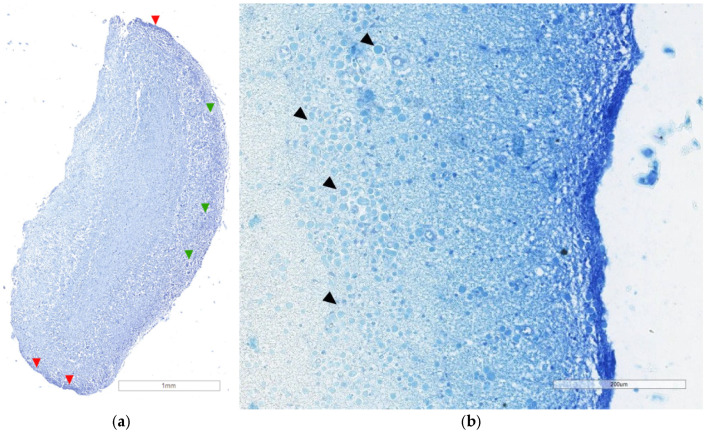
Human olfactory bulb. Nissl staining. (**a**) General view of an olfactory bulb section. The layered organization is clearly visible, particularly the glomerular layer and the layer of incoming olfactory nerve fibers (glomeruli are indicated by green arrows, and the layer of incoming olfactory nerve fibers is indicated by red arrows). (**b**) Higher-magnification image of a portion of the olfactory bulb. Clusters of corpora amylacea, occupying a substantial portion of the deeper layers of the bulb, are clearly visible (indicated by black arrows).

Nissl staining clearly demonstrated the layered structure of the olfactory bulb. Corpora amylacea were identified in some sections, where they occupied a substantial volume and were located predominantly within the internal and external plexiform layers (Figure 1a,b). Higher-resolution olfactory bulb images for this and the subsequent figures are available in the Supplementary Materials.

**Figure 2:**
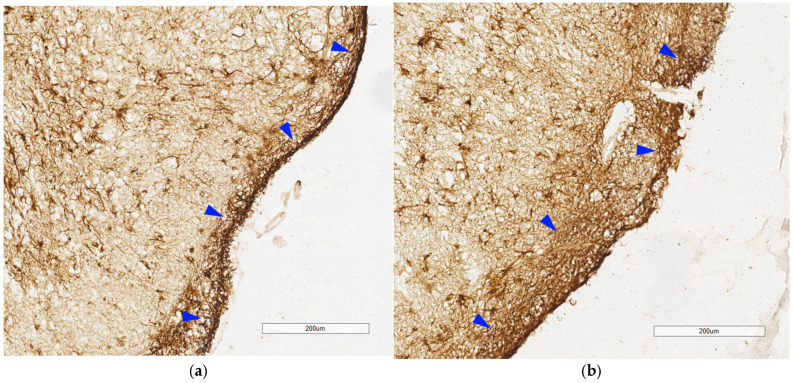
Chromogenic staining for GFAP. Representative images from both groups. The images clearly demonstrate the difference in the thickness of the olfactory nerve fiber layer (indicated by blue arrows). (**a**) Olfactory bulb of a 55-year-old patient with schizophrenia; (**b**) olfactory bulb of a 74-year-old individual with no identified nervous system pathology.

**Figure 3:**
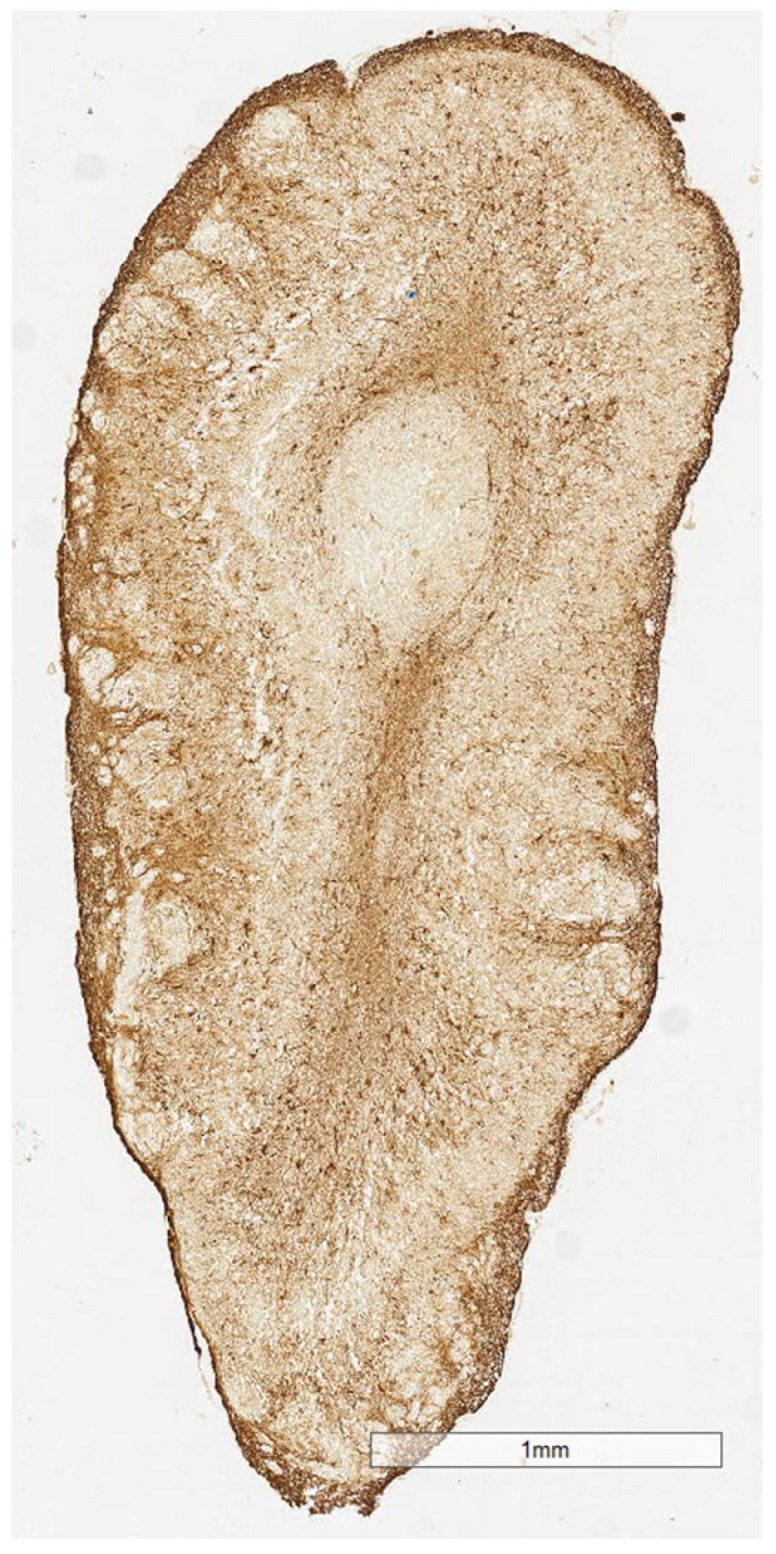
A full-size section of the olfactory bulb of 55-year-old patient suffered from schizophrenia, stained with antibodies against GFAP.

GFAP+ structures were detected in all layers of the olfactory bulb across all examined samples. The strongest immunohistochemical staining for this marker was observed in the layer of incoming olfactory nerve fibers (the first layer) and the glomerular layer (the second layer). Representative images of GFAP-stained sections from the first layer in the two groups are presented in Figure 2a,b. A representative olfactory bulb section stained for GFAP alone is shown in Figure 3. This micrograph clearly illustrates the distribution of GFAP+ structures throughout the olfactory bulb.

**Figure 4:**
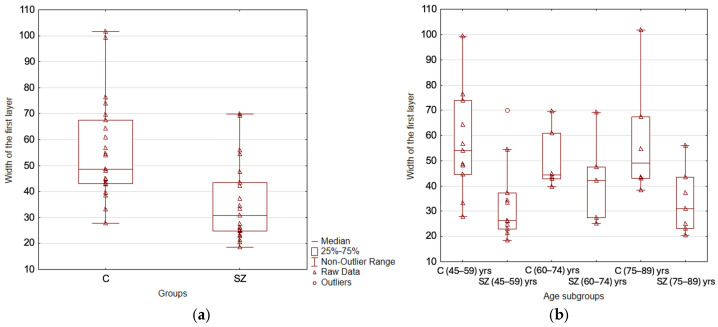
Box plots of first-layer thickness in the schizophrenia and control groups. (**a**) A statistically significant reduction in first-layer thickness was observed in the schizophrenia group. (**b**) Comparison of this parameter across age subgroups revealed a statistically significant difference only between the corresponding middle-aged subgroups of the two groups.

A significant reduction in the thickness of the first layer was observed in the schizophrenia group compared with the control group (*p* = 0.0002; Figure 4a). Comparison of this parameter across age subgroups revealed a statistically significant difference only between the corresponding middle-aged subgroups of the two groups (*p* = 0.029; Figure 4b).

**Figure 5:**
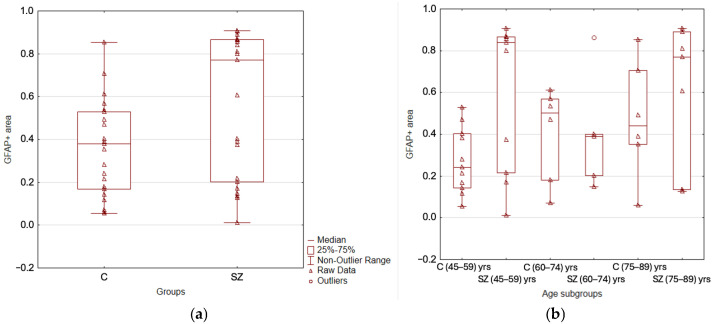
Box plots of the percentage area occupied by GFAP+ structures. (**a**) A statistically significant increase was observed in the schizophrenia group compared with the control group (*p* = 0.033). (**b**) No statistically significant differences were detected among the age subgroups within either group.

A comparison of the percentage area occupied by GFAP+ structures relative to the total section area revealed a statistically significant increase in the schizophrenia group (*p* = 0.033). However, no statistically significant differences were detected among the age subgroups within either group. The distribution of the data is presented in Figure 5a,b.

No correlation was observed between patient age and the GFAP+ area in either group (r = 0.14 and r = −0.06 in the control and schizophrenia groups, respectively).

**Figure 6:**
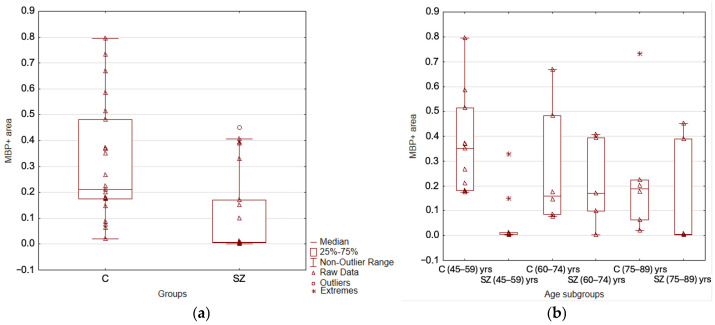
Box plots of the percentage area occupied by MBP+ structures. (**a**) A statistically significant decrease was observed in the schizophrenia group compared with the control group (*p* = 0.0002). (**b**) Comparison of the age subgroups revealed a statistically significant difference only between the corresponding middle-aged subgroups (*p* = 0.001).

A comparison of the percentage area occupied by MBP+ structures relative to the total section area revealed a statistically significant decrease in the schizophrenia group compared with the control group (*p* = 0.0002). Analysis of the age subgroups identified a statistically significant difference between the corresponding middle-aged subgroups (*p* = 0.001; Figure 6a,b).

While the middle-aged subgroup (n = 11) provides a sufficiently robust sample size for non-parametric evaluation, the statistical power for the older cohorts is inherently limited by the smaller sizes of the elderly and old-aged subgroups (n = 5 to n = 7). Consequently, the findings regarding these older age categories should be interpreted strictly as exploratory and hypothesis-generating rather than definitive.

**Figure 7:**
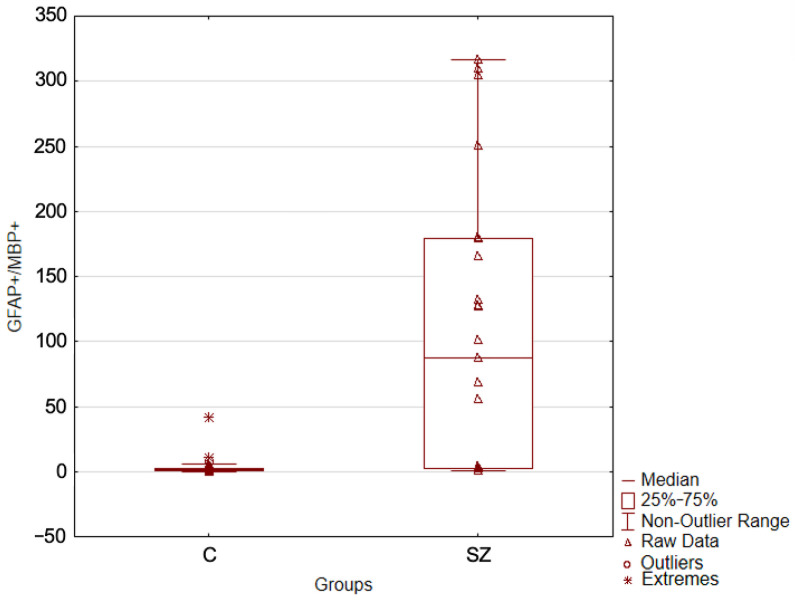
Box plot of the GFAP+/MBP+ area ratio in the schizophrenia and control groups. A statistically significant increase in this ratio was observed in the schizophrenia group compared with the control group.

**Figure 8:**
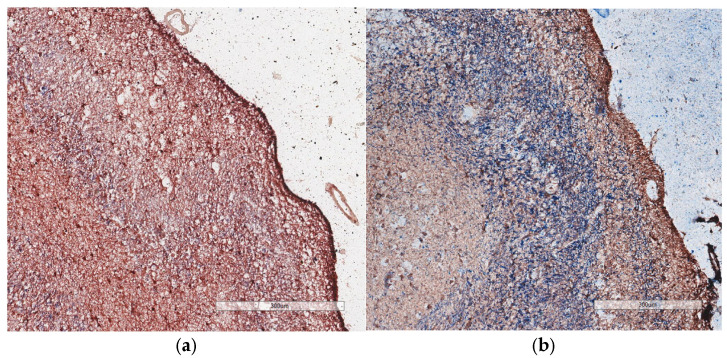
Representative images of double-stained olfactory bulb sections (GFAP, red; MBP, blue). (**a**) Olfactory bulb of an 81-year-old patient with schizophrenia. Sparse MBP staining is evident within the middle layers. (**b**) Olfactory bulb of a 57-year-old individual with no identified central nervous system pathology. GFAP staining is shown in red, with particularly intense labeling in the first and second layers. A distinct band of MBP staining (blue) is clearly visible within the middle layers.

A comparison of the GFAP+/MBP+ area ratios revealed a statistically significant increase in the schizophrenia group compared with the control group (*p* = 0.0006) when the groups were analyzed without age stratification (Figure 7). Analysis of the age subgroups demonstrated a statistically significant difference between the control and schizophrenia groups only within the middle-aged subgroup (*p* = 0.004). Representative images of double-stained olfactory bulb sections are presented in Figure 8a,b.

## 4. Discussion

Using Nissl staining, we found no differences in the overall histological organization of the olfactory bulb layers between the schizophrenia and control groups. Likewise, no visible differences were observed in the structure of the glomerular layer compared with the control group, which is consistent with the findings reported by Lise Rioux and colleagues [13]. However, toluidine blue staining lacks sufficient specificity and provides only a general overview of layer cytoarchitecture. Therefore, it is not possible to conclude that the glomerular layer is unaffected, and its structure requires further investigation using more specific markers.

A substantial number of round encapsulated structures were observed in some sections, located predominantly along the boundaries between layers. The highest abundance of these structures was detected in the deeper layers of the olfactory bulb. Based on their characteristic staining pattern, these structures were identified as corpora amylacea [70,71], also known as vasteosomes [3] or polysaccharide granules [72]. In some sections, they occupied a considerable area and could potentially disrupt the cytoarchitecture of the bulb, as well as interfere with the formation of new neural connections and the normal functioning of existing neural networks.

The primary aim of this study was to analyze the glial component of the olfactory bulb. We examined markers of astrocytes and oligodendrocytes, whereas microglia were not investigated. Among the potential markers for future studies, we plan to use the activated microglia marker Iba-1. This will provide a more comprehensive characterization of glial alterations and help identify potential neuroinflammatory processes in the olfactory bulb of patients with schizophrenia. The sections were stained using established markers for both cell types: GFAP for astrocytes and MBP for myelin produced by oligodendrocytes.

In the layer of incoming olfactory nerve fibers, GFAP is expressed primarily by two cell types: protoplasmic astrocytes [73], a subtype characterized by short, broad, and frequently branched processes, and olfactory ensheathing cells [74]. The latter exhibit properties associated with both astroglia and oligodendroglia. Therefore, the observed changes cannot be attributed exclusively to alterations in the astrocyte population.

In their review, Hans-Gert Bernstein and colleagues noted that findings regarding changes in the density of GFAP+ structures remain inconsistent [22]. However, based on the results reported by six research groups, the authors suggested that localized processes may contribute to reductions in the astrocyte population. A common limitation of many morphological studies is the relatively small sample size combined with a broad age range or the absence of age-based subgroup analyses. This issue is particularly relevant when evaluating gliosis, as age-related glial changes occur in several brain regions even in individuals without central nervous system pathology [70,71]. The review also cites studies reporting both an absence of changes [43,44] and evidence of increased numbers of GFAP+ structures [47,48,49,50].

The statistically significant increase in the area occupied by GFAP+ structures in patients with schizophrenia compared with the control group does not support the hypothesis of an astroglial deficit in schizophrenia. Instead, it suggests the presence of gliosis within this anatomical structure. This increase may be associated with enhanced astrocyte proliferation, hypertrophy of existing astrocytes, or elevated GFAP expression.

The present findings demonstrate a significant reduction in the thickness of the first layer of the olfactory bulb, accompanied by a relative increase in the area of GFAP immunopositivity in patients with schizophrenia. These observations suggest abnormalities in the spatial organization of GFAP+ structures across the layers of the olfactory bulb in this group.

Another noteworthy finding is the absence of a correlation between GFAP+ staining area and age in either the schizophrenia or control group. It is possible that the progression of gliosis with age is determined less by increases in astrocyte number or occupied area and more by hypertrophy of astrocytic processes and increased metabolic activity within existing cells. However, this interpretation remains speculative and requires confirmation through additional methodological approaches.

In contrast to astrogliosis, deficit in myelin-associated components in schizophrenia has been documented extensively and is generally regarded as a consistent finding. Accordingly, we attribute the reduction in MBP+ staining area observed in the olfactory bulb of patients with schizophrenia to a decrease in the overall oligodendrocyte population, which is consistent with findings reported in other brain regions [53,75]. However, this interpretation is based primarily on its concordance with previously published data. Precise determination of the mechanisms underlying the reduction in MBP+ immunoreactive area, specifically, distinguishing between structural demyelination and a literal decrease in cell density, will require a more comprehensive analysis that includes direct cell quantification. This objective will be addressed in future studies building upon the findings of the present exploratory investigation.

The consistency between our findings in the olfactory bulb and those reported in other brain regions suggests that the observed alterations may reflect a systemic rather than a localized process. The reduction in MBP+ area may be associated with impairment of the myelination. Within the framework of the glutamatergic hypothesis of schizophrenia, such changes may result from excitotoxic damage caused by compensatory glutamate release affecting oligodendrocytes [76].

An important parameter evaluated in this study was the ratio of GFAP+ to MBP+ staining area. The variability of this index within the schizophrenia group was 7.6-fold greater than that observed in the control group. Analysis of non-parametric effect sizes demonstrated that the observed glial imbalance was driven predominantly by the reduction in the oligodendroglial marker rather than by changes in the astrocytic marker. Specifically, the decrease in MBP+ area showed a large effect size (r = 0.54; Z = 3.69), whereas the increase in GFAP+ area exhibited a moderate effect size (r = 0.31; Z = −2.13). These findings indicate that although both pathological processes coexist, the structural deficit of the myelin component represents the more pronounced morphometric alteration within the olfactory pathways of patients with schizophrenia. Overall, a significant imbalance in glial cell composition appears to be present in a subset of schizophrenia cases.

Most statistically significant differences were observed in the middle-aged subgroup rather than in the elderly or old-age subgroups. We attribute this pattern to the accumulation of age-related changes over time, which may partially obscure disease-specific alterations. Increasing the sample size of the elderly and old-age subgroups may help clarify this issue in future studies.

Although normal data distribution was observed in several datasets, the presence of systematic deviations on the Normal P-Plot and the lack of homogeneity of variances precluded the use of parametric statistical tests. This represents a methodological limitation of the present study. In addition, the mathematical properties of the GFAP+/MBP+ ratio introduce a specific constraint. When the MBP+ area in the denominator approaches zero because of localized demyelination, the ratio may increase disproportionately, resulting in individual outliers. Although the use of rank-based non-parametric statistical methods effectively minimized the influence of these outliers on the principal findings, the inherent variability of ratio-based metrics under conditions of severe myelin loss should be considered when interpreting the results.

In addition to these statistical limitations, the use of a single representative mid-section from each sample constitutes a further constraint, as the human olfactory bulb exhibits substantial heterogeneity along its anteroposterior axis. Although intra-section variability was minimized by averaging 20 independent measurements evenly distributed around the entire perimeter of each whole-section image, future studies incorporating multi-level cross-sectional sampling will be necessary to comprehensively characterize these spatial alterations throughout the full length of the olfactory bulb.

Another important limitation of this study is the absence of precise individual postmortem interval data in the archival records. Although all autopsies were strictly regulated and performed within a standardized period of less than 24 h after death, thereby preventing substantial tissue autolysis, subtle variations in precise individual postmortem interval within this interval cannot be completely excluded and should be considered when interpreting staining intensity. Furthermore, comprehensive information regarding smoking habits was unavailable, representing a relevant limitation in studies of structures associated with olfactory function. Nevertheless, several studies have suggested that age exerts a greater influence on olfactory function than smoking status [77]. Conversely, an advantage of the present study is that all post-mortem material was collected before the emergence of the SARS-CoV-2 pandemic. This is important because SARS-CoV-2 can affect the normal functioning of the olfactory system [78], whereas reliable information regarding prior infection, particularly asymptomatic infection, is often unavailable. Future studies will expand upon the present work through the use of additional immunohistochemical markers and complementary analytical approaches.

A further methodological limitation is the difference in causes of death between the study cohorts. The control group was predominantly composed of individuals with advanced oncological diseases, often accompanied by chemotherapy exposure and terminal systemic inflammation, whereas the schizophrenia group consisted primarily of individuals who died from acute cardiovascular or respiratory events. Existing evidence indicates that tumors and cancer therapies can influence neuroglial homeostasis. While the initial astrocytic response to brain tumors is characterized by reactive gliosis, surviving neoplastic cells may subsequently alter astrocytic phenotypes. In addition, chemotherapeutic agents such as doxorubicin have been associated with increased GFAP expression [79], whereas chemotherapy-induced neuroinflammation and systemic inflammatory processes have been linked to demyelination and astrocytic reactivity [80,81]. Because these disease- and treatment-related factors may independently affect GFAP and MBP expression patterns, and their individual contributions cannot be reliably separated in a retrospective study, they should be considered important potential confounding factors when interpreting the observed differences in glial marker ratios.

Additionally, the absence of individual pharmacological histories related to antipsychotic treatment represents an unavoidable limitation of this study. Existing evidence regarding the effects of antipsychotic medications on neuroglia remains inconsistent. For example, chronic exposure to haloperidol or olanzapine in primates has been associated with a significant reduction in astrocyte numbers and a nonsignificant decrease in oligodendrocyte numbers [82]. In contrast, studies in rodent models have reported no significant changes in GFAP levels following antipsychotic treatment, suggesting that reactive astrogliosis is not simply a consequence of medication exposure [83]. Furthermore, in vitro studies have indicated that neuroleptic agents may promote oligodendrocyte differentiation and increase the expression of myelin-related proteins [84]. Because the design of the present study does not allow disease-specific alterations to be distinguished from medication-induced adaptations, these potentially divergent effects should be carefully considered when interpreting the findings.

## 5. Conclusions

In this study, we demonstrate for the first time a reduction in myelin basic protein and an increase in the astroglial marker GFAP in the human olfactory bulb, together with evidence of a potential imbalance in macroglial composition within this structure in schizophrenia. In addition to comparing the pathological and control groups, we stratified cases into age subgroups, several of which contained sufficient numbers of cases to detect statistically significant differences. Based on the overall findings, we hypothesize that the pathogenesis of schizophrenia involves, among other mechanisms, disturbances in glial population function. Such alterations may disrupt the homeostasis of the neuronal microenvironment surrounding nerve fibers and subsequently contribute to abnormalities in dendritic tree formation and dysfunction of neural networks.

## Figures and Tables

**Table 1:** Summary of autopsy OB tissue source.

Sample Group	Age Group *	Sex	Number	Age	Psychiatric Diagnosis	Cause of Death	Data Associated with Smoking
Schizophrenia (SZ)	Middle (45–59 y.o.)	Male	57-06	56	Paranoid schizophrenia	AMI	No data
			35-06	47	Paranoid continuous schizophrenia	Acute cardiomyopathy	No data
			38-06	56	Paranoid schizophrenia	PE	COPD
			29-06	59	Paranoid schizophrenia with a pronounced defect	Gastrointestinal bleeding	COPD
			39-06	53	Paranoid schizophrenia	Lobar pneumonia	No data
			13-06	59	Paranoid schizophrenia	AMI	No data
		Female	171	53	Paranoid schizophrenia	No data	No data
			28-06	56	Schizophrenia in remission	AMI	No data
			90-06	53	Paranoid schizophrenia	AMI	Stage 2 hypertension
			78-06	55	Paroxysmal-progressive schizophrenia	AMI	No data
			09-06(1)	55	Paranoid schizophrenia	Small focal confluent pneumonia	No data
	Elderly (60–74 y.o.)	Male	91-06	67	Paranoid schizophrenia with a pronounced defect	Right-sided hypostatic pneumonia	Stage 2 hypertension
		Female	25-08	68	Paroxysmal-progressive schizophrenia	AMI	Hypertension
			88-06	73	Paroxysmal-progressive schizophrenia	AMI	Stage 3 hypertension
			56-06	70	Paranoid schizophrenia	Diffuse small focal cardiosclerosis	No data
			15-06	73	Residual schizophrenia	Vascular atrophic dementia	No data
	Old (75–89 y.o.)	Male	12-12	80	Schizophrenia	No data	No data
			24-06	81	Paranoid schizophrenia	AMI	No smoking
		Female	35-06(1)	82	Paranoid continuous schizophrenia	AHF	Smoker’s bronchitis
			45-06	76	Paranoid schizophrenia	Small focal pneumonia	No data
			40-06	82	Paroxysmal schizophrenia	Diffuse small focal cardiosclerosis	No data
			09-06	81	Paroxysmal-progressive schizophrenia	Hip facture	No data
			48-06	75	Paranoid schizophrenia	Large-focal postinfarction cardiosclerosis	No data
Comparison (C)	Middle (45–59 y.o.)	Male	649-08	57	No	NHL Stage 4	No data
			877-08	56		Acute leukemia	No data
			982-06	49		Gangrene of the small intestine	No data
			1516-06	53		MDS with development of hemorrhagic syndrome	No data
			495-08	58		Colon cancer	No data
			103-08	54		Pancreatic cancer with metastases	No data
		Female	1032-08	47		No data	No data
			220-08	45		Cervical cancer	No data
			567-08	51		Brain infarction	Stage 3 hypertension
			931-08	46		No data	No data
			1800-07	47		CML	No data
	Elderly (60–74 y.o.)	Male	176-08	71		Rectal cancer	No data
			129-08	68		AHF	No data
		Female	280-08	70		Rectal cancer	Stage 3 hypertension
			862-08	74		AHF	No data
			857-08	74		Brain infarction	No data
			863-08	79		Colon cancer	No data
	Old (75–89 y.o.)	Male	1803-07	81		Right-sided lower lobe pneumonia	No data
			136-08	78		Brain infarction	No data
			1730-07	75		AMI	No data
		Female	445-08	84		Dyscirculatory encephalopathy	No data
			667-08	83		Brain infarction	COPD
			307-08	75		Bilateral abscessing pneumonia	No data

Note. AMI = Acute myocardial infarction; PE = Pulmonary embolism; COPD = Chronic Obstructive Pulmonary Disease; AHF = Acute heart failure; NHL = Non-Hodgkin Lymphoma; MDS = Myelodysplastic syndrome; CML = Chronic myelogenous leukemia. *—According to the classification of the World Health Organization (WHO).

## Data Availability

The raw data supporting the conclusions of this article will be made available by the authors on request.

## Supplementary Materials

The following supporting information can be downloaded at https://www.mdpi.com/article/10.3390/life16071053/s1, Figure S1: Human olfactory bulb. Nissl staining. (a) General view of a section of the olfactory bulb. The organization of the layers is evident, with the glomerular layer and the layer of incoming olfactory nerve fibers being particularly clearly defined (the glomeruli are shown by the green arrows, the layer of incoming olfactory nerve fibers is shown by the red arrows); (b) A magnified image of a portion of the olfactory bulb. Clusters of vasteosomes, occupying a significant portion of the deep layer of the bulb (shown by black arrows), are clearly visible; Figure S2: Chromogenic staining for GFAP. Representative images for both groups. This pair of images clearly shows the difference in the width of the olfactory nerve inflow layer (indicated by blue arrows). (a) Olfactory bulb of a 55-year-old patient suffered from schizophrenia; (b) Olfactory bulb of a 74-year-old patient with no identified pathology of the nervous system; Figure S3: A full-size section of the olfactory bulb of 55-year-old patient suffered from schizophrenia, stained with antibodies against GFAP; Figure S4: Representative images of sections of the olfactory bulb with double staining (GFAP—red and MBP—blue): (a) Olfactory bulb of 81-year-old patient with schizophrenia. Sparse staining of myelin basic protein is evident in the middle layers; (b) Olfactory bulb of 57-year-old patient with no central nervous system pathology. GFAP is stained red, with particularly strong staining in the first and second layers. A band of blue-stained MBP is clearly visible in the middle layers.

## Abbreviations

The following abbreviations are used in this manuscript:

OB	Olfactory bulb
GFAP	Glial fibrillar acid protein
MBP	Myelin basic protein
AMI	Acute myocardial infarction
PE	Pulmonary embolism
COPD	Chronic Obstructive Pulmonary Disease
AHF	Acute heart failure
NHL	Non-Hodgkin Lymphoma
MDS	Myelodysplastic syndrome
CML	Chronic myelogenous leukemia
