# Supplementary figures and images for: Neuroglia Alterations in the Olfactory Bulbs in Patients with Schizophrenia: An Exploratory Postmortem Study
Source: Life (Basel). 2026 Jun 24;16(7):1053. doi: 10.3390/life16071053 (PMC13413402; doi:10.3390/life16071053)

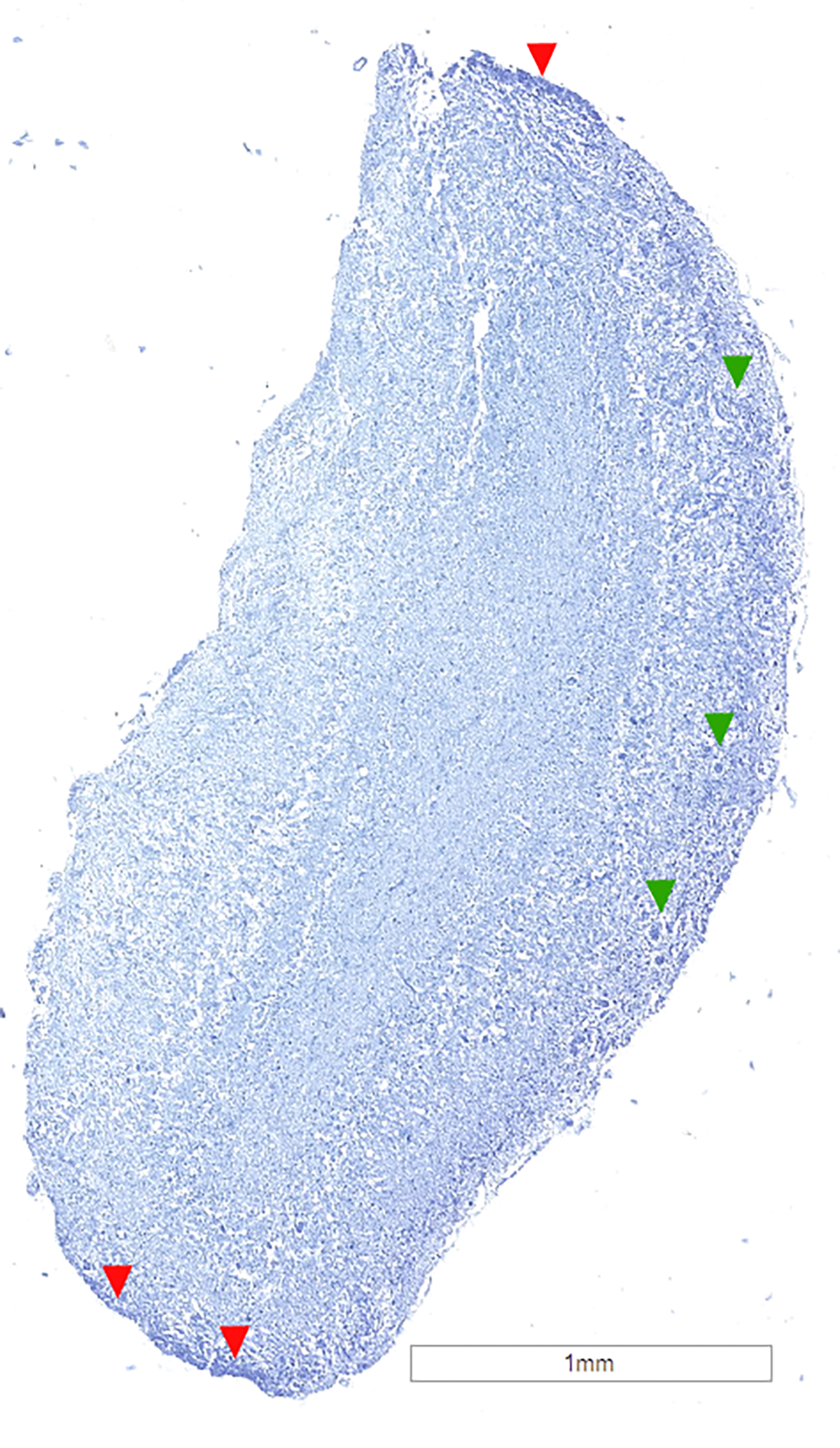

Supplement: Supplementary file 1 [file life-16-01053-s001.zip › FigS1a.png]

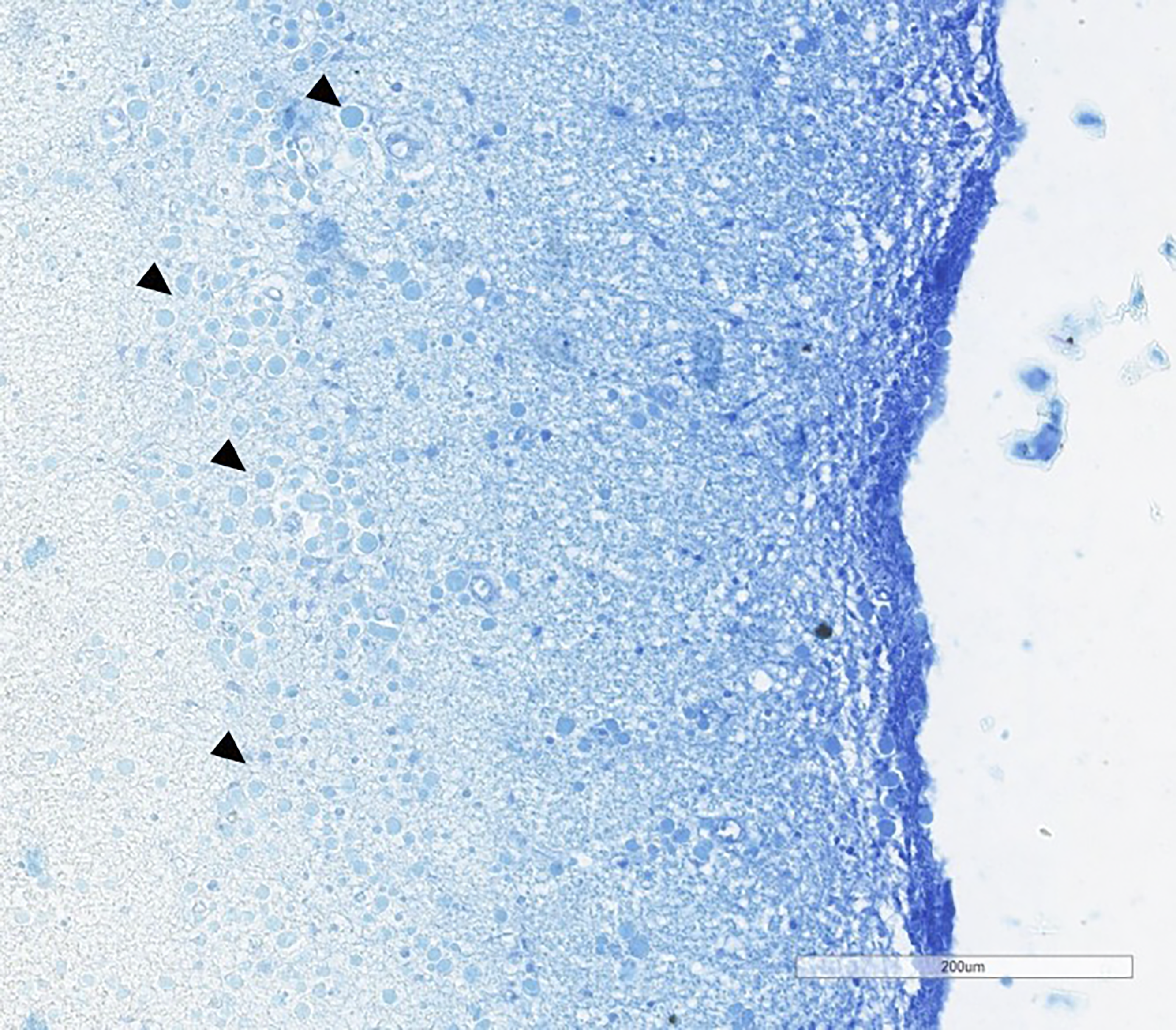

Supplement: Supplementary file 1 [file life-16-01053-s001.zip › FigS1b.png]

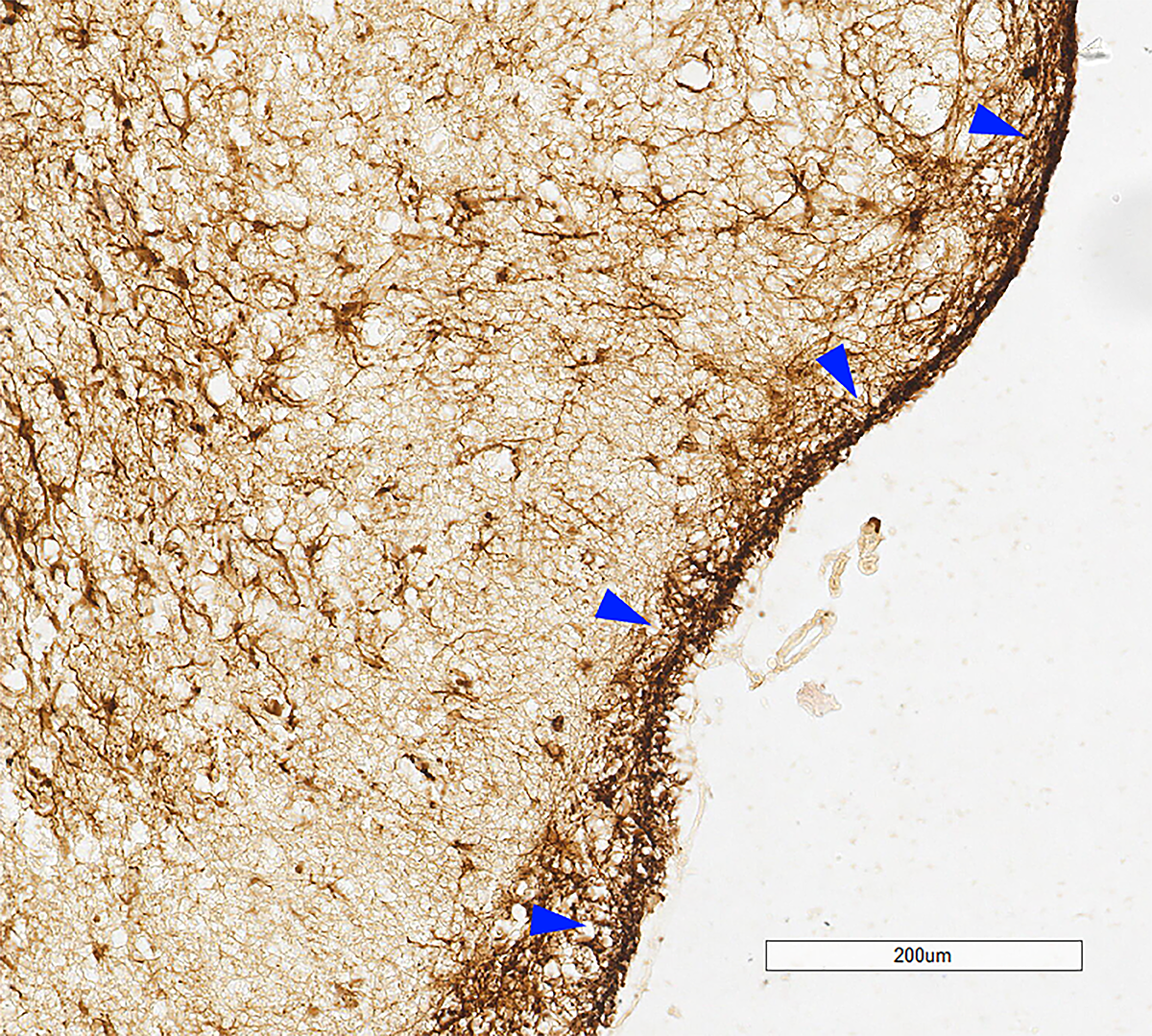

Supplement: Supplementary file 1 [file life-16-01053-s001.zip › FigS2a.png]

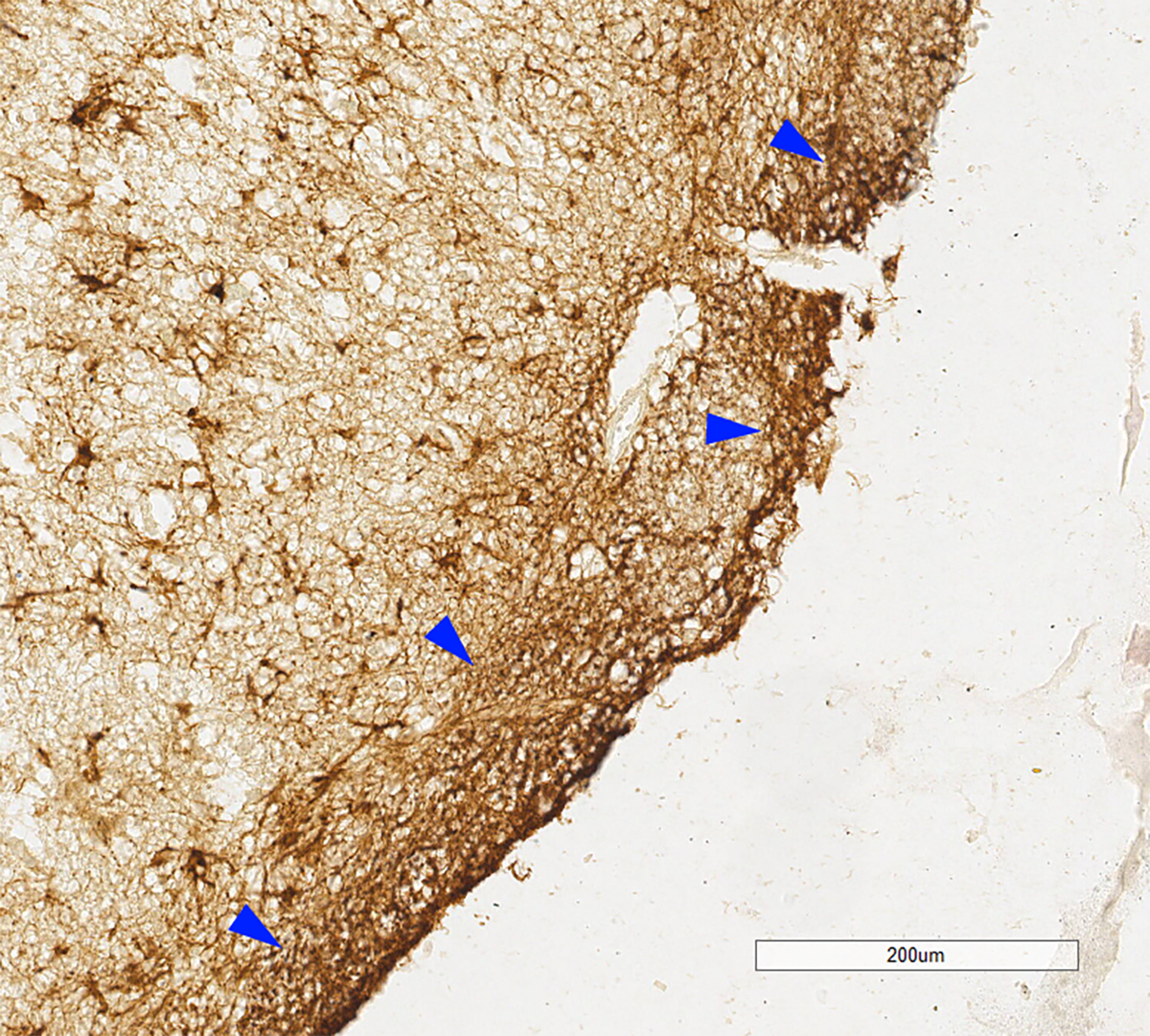

Supplement: Supplementary file 1 [file life-16-01053-s001.zip › FigS2b.png]

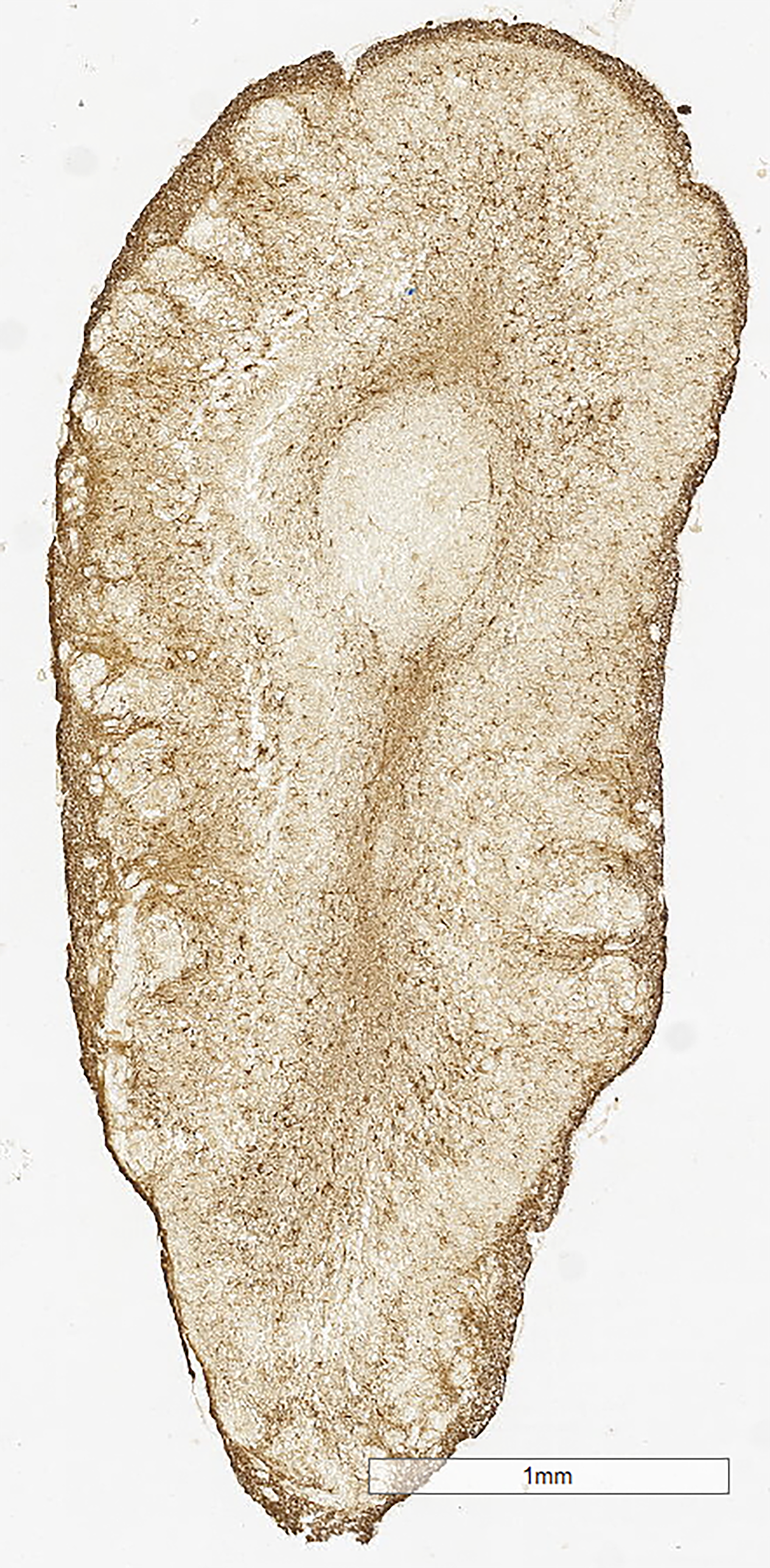

Supplement: Supplementary file 1 [file life-16-01053-s001.zip › FigS3.png]
